# Easy identification of insertion sequence mobilization events in related bacterial strains with ISCompare

**DOI:** 10.1093/g3journal/jkab181

**Published:** 2021-06-17

**Authors:** Ezequiel G Mogro, Nicolás M Ambrosis, Mauricio J Lozano

**Affiliations:** Departamento de Ciencias Biológicas, Facultad de Ciencias Exactas, IBBM—Instituto de Biotecnología y Biología Molecular, CONICET, CCT-La Plata, Universidad Nacional de La Plata, La Plata 1900, Argentina

**Keywords:** insertion sequence, transposon, genomics, Group II introns

## Abstract

Bacterial genomes are composed of core and accessory genomes. The first is composed of housekeeping and essential genes, while the second is highly enriched in mobile genetic elements, including transposable elements (TEs). Insertion sequences (ISs), the smallest TEs, have an important role in genome evolution, and contribute to bacterial genome plasticity and adaptability. ISs can spread in a genome, presenting different locations in nearly related strains, and producing phenotypic variations. Few tools are available which can identify differentially located ISs (DLISs) on assembled genomes. Here, we introduce ISCompare, a new program to profile IS mobilization events in related bacterial strains using complete or draft genome assemblies. ISCompare was validated using artificial genomes with simulated random IS insertions and real sequences, achieving the same or better results than other available tools, with the advantage that ISCompare can analyze multiple ISs at the same time and outputs a list of candidate DLISs. ISCompare provides an easy and straightforward approach to look for differentially located ISs on bacterial genomes.

## Introduction

Bacterial genomes are a mosaic composed of a core genome of housekeeping and essential genes, and an accessory genome enriched in mobile genetic elements, which can be grouped into two main classes: plasmids and bacteriophages, and transposable elements (TEs) (Siguier *et al.* 2014). Insertion sequences (ISs) are the smallest TEs, being only formed by a transposase coding region and short imperfect terminal inverted repeats (IRs), one of which (IRL) usually contains part of the transposase promoter (Mahillon and Chandler 1998; Siguier *et al.* 2014). ISs are classified by the nature of their transposases, which include DDE, DEDD, HUH, and Ser transposases (Siguier *et al.* 2017), and other features including the length and sequence of their IRs, the characteristics of the short flanking direct repeats often generated upon insertion on the target DNA (DRs—TSD, target site duplication, in eukaryotes), their genetic organization, and the target sequences into which they insert (Siguier *et al.* 2014). In addition, transposition mechanisms that generate copies of the IS (copy-and-paste), mechanisms where the IS moves from one location to another (cut-and-paste), and mechanisms where the ISs forms co-integrates, have been described (Siguier *et al.* 2014). Once an IS is acquired it can spread in a genome by transposition, and though most insertions might be deleterious, some may be beneficial and confer an increased fitness or provide a selection advantage (Schneider and Lenski 2004). One of the most important roles of ISs is their participation in genome evolution, being mediators of genomic rearrangements, gene inactivation, over-expression, and modulation of the expression of neighbor genes (Siguier *et al.* 2014). ISs contribute to bacterial genome plasticity and to the adaptability of its phenotypic traits (Schneider and Lenski 2004), such as resistance to antibacterial agents (Mugnier *et al.* 2009), virulence, pathogenicity, catabolism (Vandecraen *et al.* 2017), defense against harmful genes (Fan *et al.* 2019), and to the adaptation of bacterial strains to vaccination strategies (Pawloski *et al.* 2014; Zomer *et al.* 2018; Carriquiriborde *et al.* 2019). For these reasons, profiling IS insertion sites and their variation between related strains has gained importance.

The principal methods used for IS profiling have been chromosomal DNA hybridization (Soria *et al.* 1994; Fan *et al.* 2019), restriction fragment length polymorphism (Das *et al.* 1995) and a diversity of PCR related methods (Bik *et al.* 1996; Suzuki *et al.* 2004; Lozano *et al.* 2010). With the advent of high throughput sequencing technologies, whole genome sequences have become readily available for most microorganisms, and several tools designed to identify and annotate ISs, such as ISSaga (Varani *et al.* 2011), ISEScan (Xie and Tang 2017), Oasis (Robinson *et al.* 2012), and ISQuest (Biswas *et al.* 2015) have been developed. Furthermore, some tools for the comparison of ISs locations between bacterial strains have been developed, most of them based on the soft mapping of short reads from whole genome shotgun sequencing experiments to a reference genome (Breseq, Barrick *et al.* 2014; Transposon Insertion Finder, Nakagome *et al.* 2014; ISMapper, Hawkey *et al.* 2015; and panISa, Treepong *et al.* 2018). Some disadvantages of these programs are that they require a high genome coverage for an accurate detection of ISs, and that they are difficult to apply to the analysis of massive genomic datasets (Adams *et al.* 2016). Also, laboratories in developing countries often do not have the resources to sequence many bacterial isolates with the required sequencing depth, which makes the use of the previous programs difficult.

A complementary tool, ISseeker (Adams *et al.* 2016), was designed for the rapid and high-throughput mapping of ISs using whole genome sequence assemblies. A special case are draft genome assemblies, where contigs are typically broken at IS locations (Sohn and Nam 2018), containing partial IS sequences in one or both ends. This tool identifies the locations of a provided IS using blast (Altschul *et al.* 1990), then its flanks are extracted and mapped against a reference genome. However, the comparison to establish differentially located ISs (DLISs) must be manually done. In addition, the analysis using ISseeker requires some background knowledge of the ISs present on the test organisms, and it can only search for one IS at a time.

In order to automate the detection of IS mobilization events in related bacterial strains we developed a new program, ISCompare, which uses complete or draft genomes as query and reference and a library of ISs to find DLISs. The method is freely available in the form of open-source code, and here we validate its use via the analysis of simulated and real genomic sequences.

## Methods

### ISCompare

**Figure 1:**
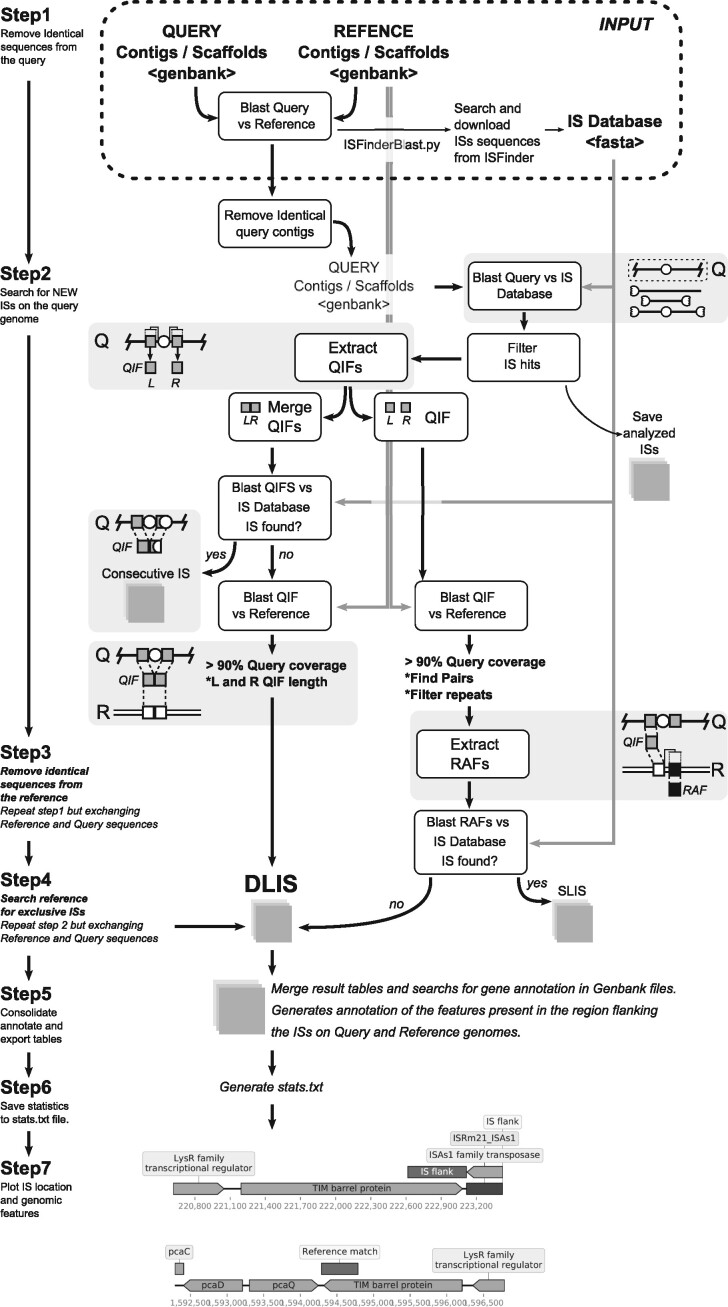
Schematic representation of the steps involved in the determination of differentially located insertion sequences by ISCompare. ISCompare requires as input Query and Reference genomes in Genbank format or their corresponding NCBI accession numbers, and optionally a database of ISs in fasta format. Step 1: blast search of the query genome against the reference genome to detect and remove identical scaffolds. Step 2: Identification of ISs using Blast and tagging of consecutive ISs. Extraction of Query IS Flanks (QIFs) and blast search of their equivalent position on the reference genome (reference anchor sequences). The QIFs are either merged or analyzed individually. In the last case, Reference Anchor Flanks (RAFs) are extracted and tested for ISs presence by blastn. Differentially located ISs (DLISs) are determined. Steps 3 and 4 are equivalent to steps 1 and 2 but exchanging the query and reference genomes. Step 5: consolidation and annotation of the results. Step 6: generation of the stats.txt file. Step 7: generation of the graphic report. Shaded boxes contain a representation of the blastn hits corresponding to ISs (white circles), the Query IS Flanking sequences (QIFs, gray boxes) to be extracted, their equivalent position on the reference genome (white boxes) determined by blastn, and the reference genome anchor flanks (RAFs, thick edge boxes). Q: query genome. R: reference genome.

An overview of ISCompare workflow is shown in Figure 1. ISCompare was written in python and uses blastn (Altschul *et al.* 1990), and Biopython (Entrez and SeqIO, Cock *et al.* 2009), DNA_features_viewer (Zulkower and Rosser 2020), Pandas (Reback *et al.* 2020), and Numpy (Van Der Walt *et al.* 2011) python modules. ISCompare takes as input query and reference genomes in Genbank flat format or its corresponding accession numbers, both of which can be either complete or draft genomes. If a list of accession numbers is provided, ISCompare downloads the genome sequences from NCBI Nucleotide databse. In addition, a library of ISs is required. This file can be provided as an optional multifasta DNA file containing the ISs, otherwise IScompare can search for the ISs present in the query and reference genomes through the use of ISFinderBlast.py script (-I option). This script uses the mechanize (Mechanize - Automate interaction with HTTP web servers. v0.4.5 2020, https://mechanize.readthedocs.io/) python module to launch an IS search at ISFinder webpage (Siguier *et al.* 2006; http://www-is.biotoul.fr). Then it collects the sequences of the found ISs into the IS.fna file located on the results folder. Once the input files have been defined the analysis begins. In the first step, a blastn search of the query genome is run against the reference genome. This step aims at removing complete identical scaffolds or replicons from the subsequent analysis. A sequence is considered identical if it has a qcovhsp (*i.e.*, query cover per high-scoring pair) greater than 99% and less than 20 unaligned nucleotides (Figure 1, step 1). With the remaining query sequences the program searches for ISs by running a blastn search against the IS database. ISCompare only makes use of the IS DNA sequences, not requiring additional information (*i.e.*, transposition mechanism, IRs, DRs, and so on). Here, blastn is run with culling_limit option set to 1 to avoid redundant hits (*i.e.*, hits from the same genomic region with different ISs on the database), and a settable *E*-value cutoff ($1.10_{\text{−}}^{10}$ by default). IS hits with an alignment length smaller than a third of the detected IS length, except for hits on scaffold ends (which on draft assemblies usually contain partial ISs), are removed. In addition, in the case of draft genomes, small query scaffolds corresponding mostly to ISs hits are removed from the analysis. Next, the sequences adjacent (500 base pairs on each side by default) to the detected ISs are extracted (Query IS Flanks—QIF), concatenated, exported as a multifasta file, and blasted against the IS database. If an IS is found on a QIF, its name is compared to the original IS, and if it is related (*i.e.*, same IS, same group, or same family), the QIF is tagged for manual verification since adjacent related ISs may produce false positives (Supplementary Figure S5, E, G, H, and J). The following step makes a blastn search of the concatenated QIFs (only those containing flanks from both sides of the IS) against the reference genome and looks for hits with high query coverage (>90%). The hits meeting this criterion correspond to ISs only present in the query genome (Figure 1, step 2). Some IS families generate short direct repeats, normally between 2 and 14 base pairs of length, upon insertion into the genome. In such cases, the small DNA sequence of less than 14 nucleotides corresponding to the DR does not interfere significantly with the blast search of the longer QIFs sequences. Next, QIFs are extracted independently and blasted against the reference genome. The hits with a query coverage greater than 90% and an E-value lower than 1.10^−10^ (default value) are taken as equivalent genomic positions in the reference genome and used as anchors to look for the presence or absence of ISs within the neighboring genomic regions (Reference Anchor Flanks—RAF; Figure 1). In this step, only the RAFs where an IS is expected (upstream or downstream) are analyzed (Figure 1, step 2). Here, several cases may arise. Some of the QIFs will match to two segments of the reference genome separated either by the length of an IS (*i.e.*, both the query and reference genome have the IS in the same location) or by a smaller distance (*i.e.*, the IS is only found on the query genome). In addition, if some QIFs contain repeated sequences, more than two matches with the reference genome will be produced. In those cases, the software tries to find the correct pairs by analyzing their location in the reference genome. If such a pair is formed, the QIFs are analyzed, else they are tagged as unspecific. In the case of draft assemblies, most of the ISs are located on scaffold ends, thus QIFs have sequences from one IS flank only. In those cases, blastn searches against the reference genome produce (ideally) only one hit. If more than one hit is produced, the QIF is tagged as unspecific. Next, the corresponding RAFs are extracted from the reference Genbank file and blasted against the IS database. If an IS of the same class or family is found, it means that the same insertion is present on both genomes, otherwise, an IS exclusively found in the query genome is informed.

In the following step, the same procedure is repeated but using the query genome as reference and the reference genome as query. This analysis reports ISs only found in the reference genome. All the results are consolidated in a single table containing the reported DLISs, the location of QIFs and reference anchor regions, and the annotation information from both the query and reference genomes. Finally, DNA_features_viewer module (-p option, Zulkower and Rosser 2020) is used to plot a PDF file with schematics of the reported ISs locations and surrounding genes. For some applications, it is useful to move the QIFs position away from the detected IS. The shift option (-S) can be used in such cases to increase the size of the region detected as an IS in a specified number of nucleotides, shifting the QIFs away (Supplementary Figures S1 and S6A).

The output of ISCompare is a set of tables and temporal raw text files. The option -c can be used to clean all the temporal files at the end of the program execution. The most important tables are: FinalResults.csv, ConsecutiveIS.csv, QueryISs.csv, RefISs.csv, and stats.txt. The FinalResuts.csv table contains the following information: IS ID, IS start, and End, Query and Reference genome IDs and the location of their ISs flanks, a field indicating if the DLIS was found on the query or reference genome, a field indicating if the IS is a DLIS or if it is recommended to verify it manually, a field indicating the quality of the blast hit (full length or partial) and the annotation of CDSs flanking the ISs. In addition, if the -p option is used, an ISGraphicReport.pdf file is generated, showing the genome context of the ISs and their flanks (Figure 1, step 7 and Figure 4B were constructed using the plots generated by ISCompare). A detailed protocol on how to run ISCompare and interpret the results is available on protocols.io: https://dx.doi.org/10.17504/protocols.io.bst6nere

The program can be run on computers with low resources. All the tests were run on common laptop computers, and on a virtual PC running with three CPUs and 3 GB of RAM. For command line options see the ISCompare GitHub page (https://github.com/maurijlozano/ISCompare).

### ISsimulator

To generate simulated genome sequences with random IS insertions, a new script ISsimulator.py, was used. ISsimulator was written in python and requires Biopython (Cock *et al.* 2009), a reference genome in Genbank format and the IS to be inserted in fasta format. The program inserts a selected number of copies of the IS at randomly chosen genomic locations, and outputs modified Genbank and fasta files, and a table with the location of the inserted ISs. ISsimulator can also create target-site duplications of a specified size.

### Sequences used on this work

All the genome sequences used in this study were downloaded from the NCBI or EMBL-EBI genome databases. The corresponding accession numbers are listed in Supplementary Table S1. A compilation of IS sequences downloaded from https://github.com/thanhleviet/Isfinder-sequences was used as IS database, except in the cases where ISCompare was used to look for a single IS.

### Average Nucleotide Identity and digital DNA–DNA hybridization analysis

Average Nucleotide Identity (ANI) and digital DNA**–**DNA hybridization (dDDH) analysis were performed at the ANI/AAI-Matrix Genome-based distance matrix calculator (Goris *et al.* 2007, http://enve-omics.ce.gatech.edu/g-matrix/) and the GGDC Genome-to-Genome Distance Calculator 2.1 (Auch *et al.* 2010, http://ggdc.dsmz.de/ggdc.php#) web servers. Phylogenetic trees were visualized with ITOL (Letunic and Bork 2019) and, when necessary, edited with Inkscape (v1.0, inkscape.org).

### Statistical analysis and figures

All the statistical analyses and the corresponding plots were done with R software (R Core Team 2014). Final figures were edited with Inkscape (v1.0, inkscape.org).

### Benchmark

Benchmark values were taken from 10 independent runs of ISSeeker or ISCompare using the Linux bash usr/bin/time command. Both programs were used to search for changes in the location of a single IS on *Ensifer meliloti* 1021 *vs* GR4 and *P. aeruginosa* PAO1 *vs* CMC-115. In addition, ISCompare was also run to search for DLISs using the complete ISFinder database (ca. 6000 ISs). The benchmark was done using a laptop computer with an Intel(R) Core (TM) i3-5010U CPU (2.10 GHz) and 4GB (1600 MHz) of RAM.

### Data availability

All the data necessary for confirming the conclusions of the article are present within the article, figures, and tables. Sequence data is available at GenBank and EMBL-EBI portals and the accession numbers are listed in Supplementary Table S1. ISCompare program can be downloaded from https://github.com/maurijlozano/ISCompare. A detailed protocol on how to run ISCompare and interpret the results is available on protocols.io: https://dx.doi.org/10.17504/protocols.io.bst6nere

All the supplementary material is available at the GSA Figshare portal. File description: Supplementary Table S1: Genomes used for ISCompare evaluation. Supplementary Table S2: SurroundingLen parameter optimization. Supplementary Table S3: ISCompare evaluation using 3000 random IS*30* insertions. Supplementary Table S4: Analysis of differentially located ISs on *P. aeruginosa* strains. Supplementary Table S5: Analysis of differentially located ISs on *E. meliloti* strains. Supplementary Table S6: Comparison of ISCompare results using the normal *vs* the Shift mode (-S). Supplementary Table S7: Comparison of ISCompare and ISSeeker using *E. meliloti* genomes. Supplementary Table S8: Comparison of ISCompare and ISSeeker using *P. aeruginosa* genomes. Supplementary Table S9: Comparison of *B. pertussis* TOHAMA I with strains I127, J299 and J412 containing a IS*481* insertion on the pertactin autotransporter gene. Supplemental File S1: ContigBlastHit.pm. Modified ContigBlastHit.pm python module from ISSeeker. Supplementary Figure S1: *E. meliloti* Average Nucleotide Identity (ANIb) matrix and UPGMA distance tree. Supplementary Figure S2: Sensitivity and precision of ISCompare using different SurroundingLen values. Supplementary Figure S3: Phylogenetic tree of all the sequenced *E. meliloti* strains at NCBI genomes database. Supplementary Figure S4: Phylogenetic tree of all the sequenced *P. aeruginosa* strains at NCBI genomes database. Supplementary Figure S5: Schematic representation of the possible cases resulting in DLIS, SLIS, and VD reports. Supplementary Figure S6: ISCompare shift mode. Supplementary material is available at figshare: https://doi.org/10.25387/g3.14573460.

## Results

### ISCompare parameter optimization

In order to optimize the program parameters, we generated artificial *Eschericia coli* str. K-12 substr. MG1655 and *E. meliloti* 2011 genomes with 100 random IS*30* or IS*Rm5* insertions, respectively and run ISCompare using different settings in a stepwise mode (Supplementary Table S2). We made two kinds of comparisons: in the first case, we compared the *E. coli* str. K-12 substr. MG1655 genome with the artificially generated one (*i.e.*, identical genetic background); in the second case we compared *E. meliloti* 1021 with the artificially generated *E. meliloti* 2011-100IS genome. *E. meliloti* 1021 and *E. meliloti* 2011 are very closely related but not identical strains, which make them good testing subjects (Supplementary Figure S1).

ISCompare was run with different surroundingLen [-s option] (*i.e.*, the length of sequence to extract from each side of a detected IS) values ranging from 100 to 2000 nucleotides in steps of 100. ISCompare outputs a results table with the detected differentially located ISs under the “DLIS” category for the most confident cases, and within several categories of “Verify manually_**…**_” or “Discarded from the analysis_**…**_” cases (Supplementary Figure S5; VD for now on) which may result in false positives, as a consequence of repeated genomic regions, consecutive identical (or very similar) ISs or when there is not a significant blastn hit for the QIFs on the reference genome. In the comparison of *E. coli* K12 genomes, where the genetic background is identical, a precision of 100% (93%) and a sensitivity of 91% (99%) were achieved for surroundingLen in the range of 200–500 nucleotides for the DLIS category. Values in parenthesis were obtained by taking the VD category as DLIS (Supplementary Figure S2). When the *E. meliloti* strains were compared, it was not possible to achieve a precision and a sensitivity greater than 90% for both DLIS and VD categories for a single surroundingLen. For DLIS results, the best surroundingLen was in the range of 400–1100 nucleotides with a precision of 98.9% and a sensitivity of 94%, while for VD the best surroundingLen was in the range from 500 to 2000 nucleotides (Supplementary Figure S2) with the best results for 1400 with a sensitivity of 100% and a precision of 86%. In general, a surroundingLen greater than 300 is recommended since lower values would decrease the Blast search sensitivity. On the other hand, values bigger than 1000–2000 base pairs could either slightly improve the detection of DLISs, or generate a greater number of false negatives, product of the overlap of the IS flanks with adjacent ISs. Thus, for the optimization of the remaining parameters, a surroundingLen of 500 nucleotides was used as it produced, in general, good results in all the analyzed cases.

Using the optimal surroundingLen the minAlnLength (*i.e.*, the minimal required alignment length of the QIFs to the reference genome), minlength (*i.e.*, the minimal QIFs length to be extracted from the genome Genbank file), ISdiff (*i.e.*, the minimal difference between the blast alignment length and the query length, which is used to discard scaffolds that only match to IS sequences) and scaffoldDiff (*i.e.*, the maximum number of allowed nucleotides missing on the blastn alignment between the query and reference genomes) were optimized by manually assigning different values. The minAlnLength and minlength arguments are more relevant for the analysis of draft genomes, where, in the case of small contigs, there might be QIFs with high query coverage, but with a length smaller than surroundingLen. Minlength option is used in such cases to filter QIFs with length minor to minlength, while minAlnLength is used to filter QIFs with a blastn alignment to the reference genome of a length smaller than minAlnLength. To optimize these parameters, we searched for draft genome assemblies nearly related to *E. meliloti* 1021 and compared them with the reference *E. meliloti* 1021 strain genome (Supplementary Figure S3). *E. meliloti* strain USDA1022 was used (Supplementary Table S1). For the assessment of true positives, the results were manually revised since no previous reports were made on the IS distribution on this strain (Supplementary Table S2). The optimal values (minLength = 50, minAlnLength = 50, ISdiff = 50, and scaffoldDiff = 20) were established as the default for the program.

### Detection of IS location changes using artificially generated genomes

To assess the sensitivity and precision of our software we compared the *E. coli* str. K-12 substr. MG1655 genome with artificial genomes on which random IS insertions were made using ISsimulator script. In addition, we used an IS database containing all the ISs found by ISFinder web server. A total of 3000 IS insertions of IS*30* were simulated in steps of one hundred per genome and analyzed using ISCompare (*i.e.*, the program was run 30 times). Our method demonstrated to have a very good recall (94%) and precision (100%), with no false positives and few false negatives (181 over 3000 positives) when analyzing the DLIS category (Supplementary Table S3). The program almost reached 100% recall (99.1%) at the expense of precision (92%) when considering as true the VD categories. It should be noted that in the case of *E. coli* str. K-12 substr. MG1655, on average, every 100 differential ISs, 12 sequences were tagged for manual inspection, which could be easily done with the -p option producing a graphic pdf report of the ISs genomic context on both the query and reference genomes. In general, the ISs tagged for manual inspection represented false positives cases (40%) mostly due to consecutive ISs (Supplementary Table S3).

IScompare can be also run to look for a specific IS (Supplementary Table S3). In such a case the results for the simulated data were similar. This was expected due to the capacity of ISCompare to distinguish consecutive insertions of different ISs.

### Comparison of *E. meliloti* 1021 and *Pseudomonas aeruginosa* PAO1 with strains with progressively decreasing digital DNA–DNA hybridization

**Figure 2:**
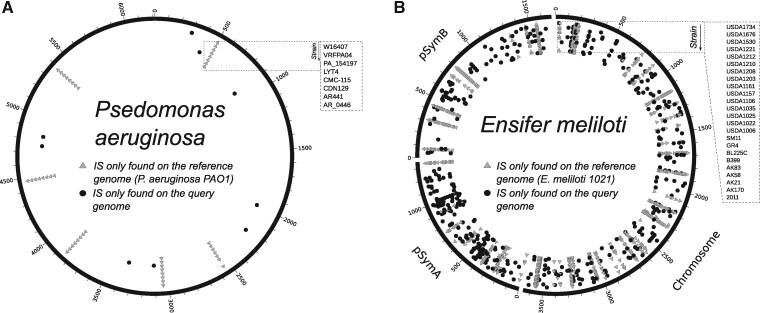
Genomic distribution of DLISs. Circos plot showing the genomic location of DLISs in *P. aeruginosa* and *E. meliloti* strains. (A) DLISs found in *P. aeruginosa. P. aeruginosa* PAO1 was used as reference and compared to strains AR_0446, AR441, CDN129, CMC-115, LYT4, PA_154197, VRFPA04 and W16407, shown in that order from the border to the center. (B) DLISs found on *E. meliloti. E. meliloti* 1021 was used as reference and compared to strains 2011, AK170, AK21, AK58, AK83, B399, BL225C, GR4, SM11, USDA1006, USDA1022, USDA1025, USDA1035, USDA1106, USDA1157, USDA1161, USDA1203, USDA1208, USDA1210, USDA1212, USDA1221, USDA1530, USDA1676, and USDA1734, shown in that order from the border to the center. Light-gray triangles: DLISs found on the reference genome. Black circles: DLISs found on the query strains.

In order to test ISCompare with real genomic data, we selected different strains of *P. aeruginosa* with complete closed genomes and with progressively decreasing values of digital DNA**–**DNA hybridization (dDDH). Ten strains meeting this criterion were selected and *P. aeruginosa* PAO1 was used as reference (Supplementary Figure S4 and Table S4). The results were manually analyzed to assess for true/false positives cases. Only the cases where an IS was found either on the reference or query genomes, and which flanks were perfectly conserved (*i.e.*, equal genomic context) were considered as true positives. On average, 4.6 DLISs were found on each genome (6.3 for the VD reports), with a maximum of 9 and a minimum of 0. The precision was very good (100%) for the DLIS category but was greatly diminished (29%) when the VD cases were taken as positive (Supplementary Table S4). It should be noted that most of the VD cases are only reported with the objective of improving the sensitivity after a manual inspection, since some true positives are usually tagged for manual verification due to repetitions and consecutive ISs. A significant correlation between dDDH and the number of discarded QIFs was found (*R=−*0.82*, P*** ***=*** **0.012), since, as it was expected, more QIFs were discarded in the filtering steps when the compared genomes were phylogenetically more distant. The distribution of the DLISs mapped to the reference genome is shown in Figure 2A.

**Figure 3:**
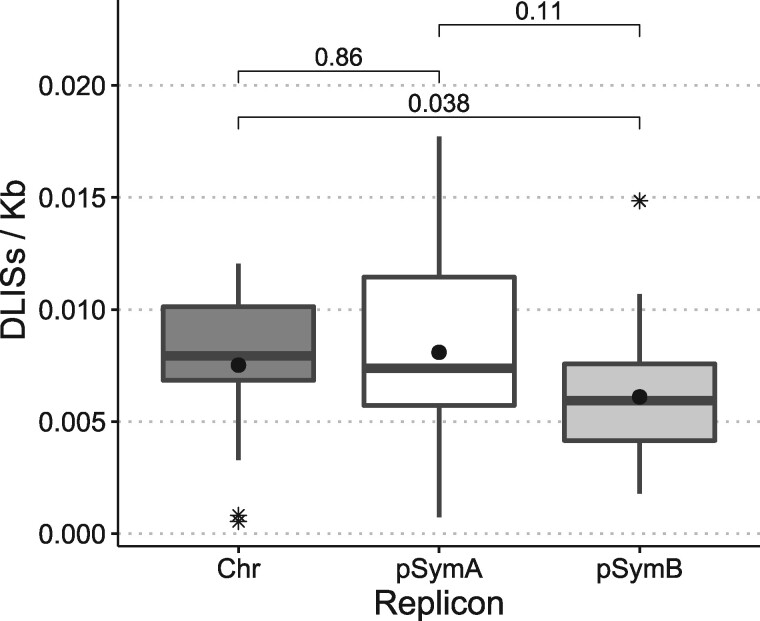
Distribution of DLISs in *E. meliloti* replicons. Boxplot of the number of DLISs per kilobase for the three replicons of *E. meliloti* in the 26 strains used. Chr, Chromosome; pSymA, megaplasmid pSymA; pSymB, megaplasmid pSymB. Asterisks: outliers. Black circles: average. The statistical analysis was performed with R. *P*-values were calculated using the Kruskal–Wallis test.

As very few DLISs were found in the case of *P. aeruginosa*, ISCompare was used to identify DLISs in *E. meliloti*, which is known to have a greater number of ISs (Sallet *et al.* 2013). Twenty-six *E. meliloti* strains were compared to *E. meliloti* 1021 using ISCompare (Supplementary Figures S1 and S3 and Tables S1 and S5). The results were manually analyzed to assess for true/false positive cases, since no previous report comparing the distribution of IS in these strains could be found. We considered as true positive only the cases where the genomic context was the same on both sides of the IS. Our software found an average of 40 DLISs on *E. meliloti*, with values fluctuating between 0 (strain 1021–2119 comparison) and 86 (strain 1021-USDA1212) (Figure 2B). In all the analyzed strains a total of 632 (27), 263 (11), and 226 (10) DLISs were located on the chromosome, the symbiotic plasmid pSymA and the chromid pSymB, respectively. On parenthesis the average number of DLISs per replicon and strain is shown. The density of DLISs per replicon was found to be higher for the symbiotic plasmid pSymA, followed by the chromosome and the chromid pSymB (Table 1), although no significant difference (*P* < 1.10^−3^) was observed (Figure 3).

**Table 1:** Distribution of DLISs on *E. meliloti* replicons

Replicon	Total DLISs	Avg. DLISs	Density		
			Total DLISs/kb	Avg. DLISs/kb	**ISs/Kb** ^a^
Chromosome	632	27	0.172	0.0075	0.013
pSymA	263	11	0.194	0.008	0.019
pSymB	226	10	0.134	0.0061	0.008

^a^ Evaluated in the reference genome.

In general, the precision was relatively stable (89% on average for DLIS, Supplementary Table S4) not showing a significant correlation with the phylogenetic distance. However, a significant correlation of dDDH with the total number of discarded sequences was found (***ρ ***=** **−0.44, *P*** ***=*** **0.032 for all the analyzed genomes and ***ρ*** = −0.8920158, *P*** ***=*** **0.00052 for the complete genomes). In the case of complete genomes, the precision was more variable ranging from 66 to 100%. This could have been due to the presence of regions with consecutive ISs, repeated sequences or rearranged genomic segments, which in complete genomes can generate false positives, while in the case of draft genomes, these regions are present on scaffold ends and usually taken as true DLISs. Another source of false positives was related to mobile group II introns. Mobile group II introns are ribozymes and retroelements present in most *E. meliloti* strains and which natural target site lies within ISs (IS*Rm2011-2* in the case of RmInt1 and on the left and right IRs of IS*Rm17* in the case of RmInt2; Toro *et al.* 2018). To avoid the errors in the detection generated by group II introns, comparisons between *E. meliloti* 1021 and GR4 strains were run using the -S (Shift) option with values in the range of 100 to 5000. For -S values between 100 and 2000 the results were the same, but with values ranging from 3000 to 5000 the results showed an improvement in the identification of DLISs, including now the partial ISs flanking group II introns (Supplementary Figure S6B). Thirty new DLISs were detected, 23 corresponding to *ltrA* (Group II intron-encoded protein Ltr, a multifunctional protein that promotes group II intron splicing and mobility) flanking ISs and 7 corresponding mostly to multiple consecutive ISs. Nevertheless, 4 DLISs that were correctly detected in the normal mode were missing (Supplementary Table S6).

In addition, ISCompare was run using as IS database a multifasta file containing all the *ltrA* gene variants found on *E. meliloti* to compare the *E. meliloti* GR4 and 1021 genomes. A surroundingLen of 500 base pairs and a shift of 4000 base pairs were used (Supplementary Figure S6C). In this comparison, a total of 6 *ltrA* genes were found on *E. meliloti* 1021. Two *ltrA* genes presented the same location, with some minor differences, in both strains; 2 were correctly detected as differentially located; and 2 that were present only on the reference strain, were tagged as discarded. This error was produced by differences in the genomic context surrounding *ltrA*.

For *E. meliloti* GR4, 21 *ltrA* genes were detected, 2 were in the same location in both strains, and of the remaining, 6 were discarded because they were in a different genomic context, 12 were correctly reported by ISCompare as differentially located, and 1 was manually detected in the discarded QIFs. In all cases, the introns that failed to be correctly reported presented differences in the genomic context, especially in differentially located ISs.

In addition, a comparison of *E. melioti* GR4 with *E. meliloti* G4 (Toro *et al.* 2016) (Supplementary Table S1) was done. G4 raw reads were downloaded from SRA (SRR2078187), assembled using SPAdes (Bankevich *et al.* 2012) and annotated with Prokka (Seemann 2014) at Galaxy Australia web server (Afgan *et al.* 2016, https://usegalaxy.org.au/). In that case, ISCompare found 6 DLISs, including all the previously reported by Toro *et al.* (2016), except a difference in a group II intron. This was most likely due to the difficult assembly of regions containing group II introns.

### Detection of IS*481* within the pertactin gene of *Bordetella pertussis*

**Figure 4:**
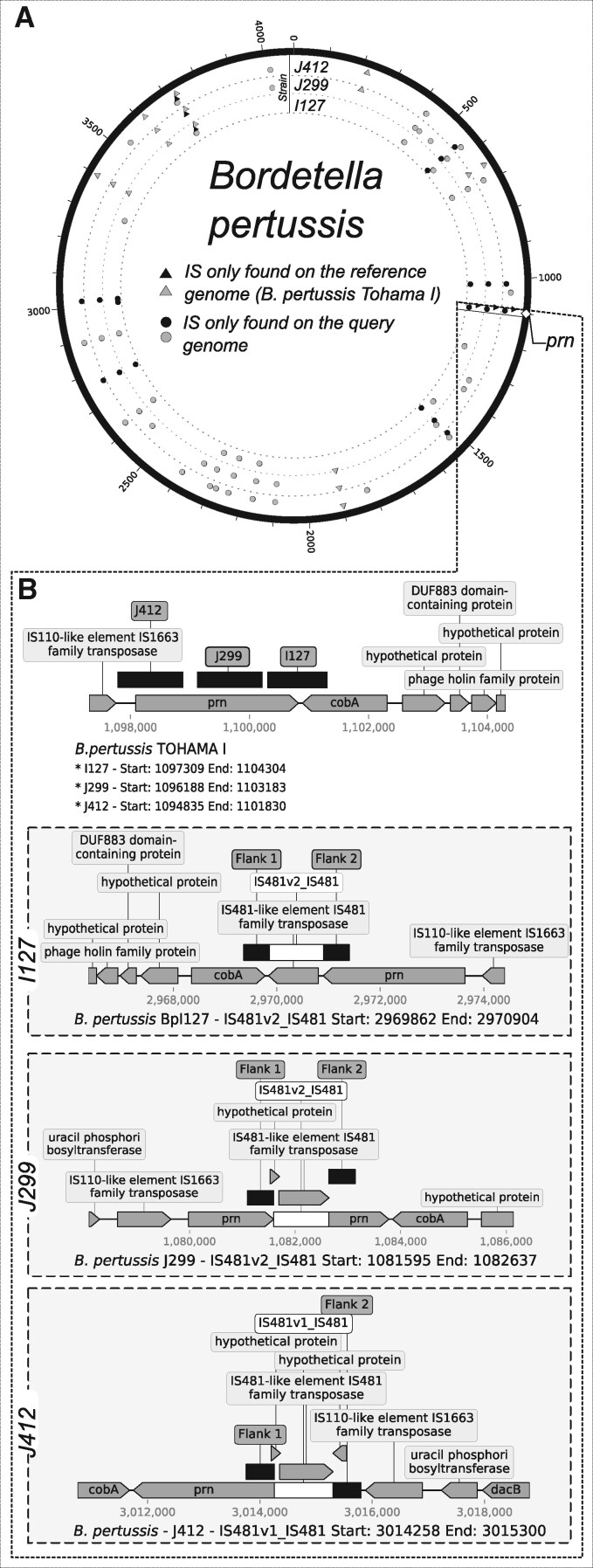
Genomic distribution of DLISs in *Bordetella pertussis* strains with an IS*481* insertion within the pertactin gene. (A) Circos plot showing the genomic location of DLISs in *B. pertussis*. *B. pertussis* TOHAMA I was used as reference and compared to strains I127, J299, and J412. (B) Adaptation of ISCompare graphic report showing the IS*481* insertion within the pertactin gene. Light-gray triangles and circles: DLISs identified by manually analyzing the VD category. Black circles and triangles: DLIS category. Circles: DLISs detected in the query genome. Triangles: DLISs detected in the reference genome.

*B. pertussis* is the causative agent of whooping cough, which has re-emerged as a public health threat despite broad vaccine coverage. The re-emergence of this pathogen has been correlated with the transition from the use of whole-cell pertussis vaccines to acellular component vaccines (Bart *et al.* 2014; Melvin *et al.* 2014) which usually contain up to 5 purified *B. pertussis* antigens, namely the pertussis toxin, (PT), Filamentous Hemagglutinin (FHA), Pertactin (PRN), and Fimbriae (FIM2 and FIM3) (Dewan *et al.* 2020). Among these, pertactin, a highly immunogenic outer membrane protein that promotes adhesion to tracheal epithelial cells (Inatsuka *et al.* 2010), has been implicated in vaccine-driven evolution presenting diverse types of knock-out mutations in circulating strains. One of the most common mutations is the insertion of IS*481* within its coding sequence, presenting at least three different locations (Pawloski *et al.* 2014). We used the IS*481*-interrupted *prn* sequences KF804023.1, KC445198.1, and KC445197.1 to look for representative genomes at the NCBI Assembly database. Three representative genomes (Supplementary Table S1.) corresponding to *B. pertussis* strains I127, J412, and J299 were analyzed with ISCompare using *B. pertussis* Tohama I as reference. ISCompare correctly identified an IS*481* insertion within the pertactin gene in *B. pertussis* strains J299 and J412, and the IS*481* insertion at the 3’- end of strain I127 pertactin gene (Figure 4B.). In addition, 7 shared DLISs were found in all the genomes (Figure 4A. Black circles and triangles; Supplementary Table S9). After a manual verification using the graphic report, 31 and 42 DLISs were found for I127 and J299/J412, respectively (Figure 4, gray circles and triangles), mainly involving cases on which two consecutive IS*481* were detected in one genome, while only one was found in the other.

### Comparison with ISseeker

Although there are several tools for the comparison of insertion sites between bacterial strains, most of them are based on the soft mapping of short reads from whole genome shotgun sequencing experiments to a reference genome (Breseq, Barrick *et al.* 2014; Transposon Insertion Finder, Nakagome *et al.* 2014; ISMapper, Hawkey *et al.* 2015; and panISa, Treepong *et al.* 2018). The only one with features similar to those of ISCompare is ISSeeker (Adams *et al.* 2016).

**Figure 5:**
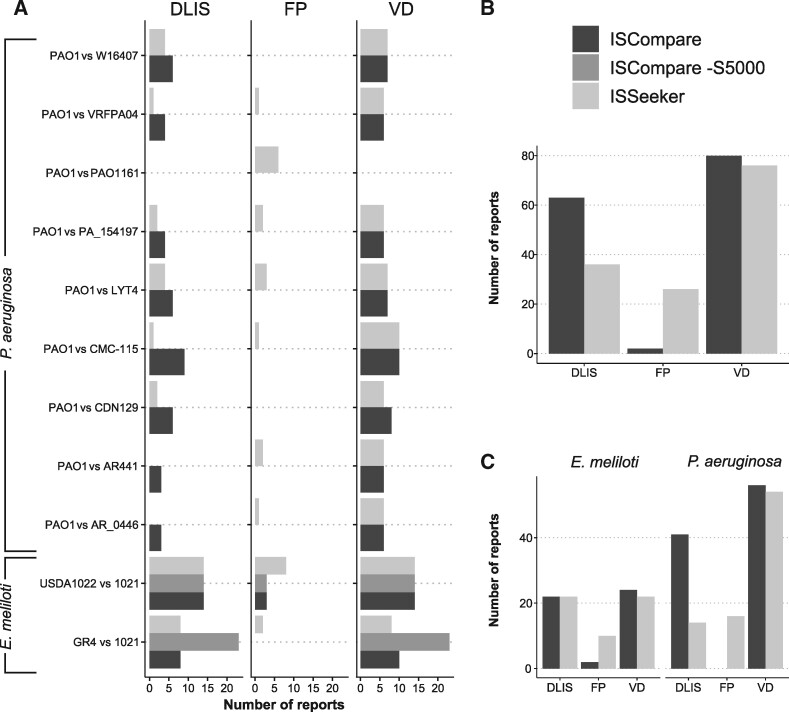
Performance of ISCompare in comparison with ISSeeker. ISCompare was compared with ISSeeker using selected ISs from *E. meliloti* and *P. aeruginosa* strains. (A) Number of DLIS, VD and FP reports for *P. aeruginosa* and *E. meliloti* strains. (B) Total number of DLIS, VD and FP reports. (C) Total number of DLIS, VD and FP reports discriminated by species. VD: Total identified DLISs, including those that were identified manually. FP: false positives. DLIS: Differentially located IS reports. In the case of ISSeeker, only ISs with assigned mates were considered as DLISs. Black: ISCompare; gray: ISCompare with -S 5000 option; light gray: ISSeeker.

To compare the performance of ISCompare with ISseeker we analyzed the location of the IS*Rm2011-2* on *E. meliloti* GR4 and 1021 using both programs (Figure 5; Supplementary Table S7). ISseeker outputs a table with the mapping coordinates of left and right query IS flanks relative to the reference genome. In addition, it attempts to find mates by comparing the mapping coordinates of all the flanks. Thus, for the analysis of ISSeeker results, we considered as DLISs those ISs which flanks were correctly mapped and mated. All the reported flanks and ISs were manually analyzed to define whether they were DLISs or not. Our program performed similarly to ISSeeker (Figure 5A), both correctly detecting 8 DLISs, and several ISs located in a different genomic context. However, ISSeeker reported ISs which were discarded by ISCompare because they contained flanks that produced blastn matches with multiple genomic regions, and in most cases corresponded to false positives. In addition, ISSeeker failed to detect *ltrA* related DLISs (15 ISs) which were detected by ISCompare using the shift option (Figure 5A, gray bars).

We also tested both programs for the identification of DLISs in the case of draft genomes. We tried to run ISSeeker for the comparison of *E. meliloti* USDA1022 (157 scaffolds) with *E. meliloti* 1021. In that case, an error occurred and ISSeeker code had to be modified (ContigBlastHit.pm; Supplemental File S1) to include ISs found on scaffold ends. The results were fairly comparable for both programs (14 DLISs were found), with ISCompare producing fewer false positives (Figure 5A; Supplementary Table S7). When we run the same comparison in the shift mode, a total of 14 (3 new) DLISs were found, but three of the previously found DLISs were missing (Supplementary Table S7).

We also tested ISSeeker with the *P. aeruginosa* dataset. In that case, the performance of ISCompare was better than that of ISSeeker, and less false positives were produced (Figure 5, A and C; Supplementary Table S8). From these results we can conclude that ISCompare performed better than ISSeeker (Figure 5B.), achieving slightly better sensitivity and precision.

Finally, we compared the performance of ISSeeker and ISCompare (Table 2). As the results show, although ISSeeker is faster and uses less memory and CPU, the resources used by ISCompare are also low, and less time is required to analyze the results. In addition, ISCompare can search for thousands of ISs simultaneously, requiring in the case of all the ISs from ISFinder (ca. 6000 ISs) only 0.04 seconds per IS.

**Table 2:** ISCompare and ISSeeker benchmark results

			Time (seconds)	Time (seconds)/IS	%CPU	RAM (MB)
*E. meliloti* 1021 *vs E. meliloti* GR4	IS*Rm2011-2*	ISseeker	4.67 (±0.09)	—	98.75 (±0.79)	59.25 (±0.03)
		ISCompare	51.61 (±0.66)	—	106.90 (±0.74)	198.33 (±0.22)
	IS database from ISFinder	ISCompare	253.49 (±8.21)	ca. 0.04	137.10 (±1.37)	198.86 (±0.39)
*P. aeruginosa* PAO1 *vs*. *P. aeruginosa* CMC-115	IS*Pa11*	ISseeker	3.59 (±0.22)	—	98.50 (±0.62)	72.54 (±0.02)
		ISCompare	37.30 (±0.46)	—	109.60 (±0.70)	210.67 (±0.28)
	IS database from ISFinder	ISCompare	240.72 (±12.06)	ca. 0.04	163.10 (±1.60)	210.78 (±0.26)

Benchmark values were taken from 10 runs comparing *E. meliloti* 1021 *vs* GR4 and *P. aeruginosa* PAO1 *vs* CMC-115 using both software (ISseeker and ISCompare) and the usr/bin/time linux bash command. time: Elapsed real time in seconds, %CPU: calculated as (CPU-time spent in kernel mode + CPU-time spent in user mode)/elapsed real time, RAM: average resident set size of the process in Megabytes.

## Discussion

ISCompare is a tool for the identification of DLISs which uses blastn (Altschul *et al.* 1990) for the pairwise genomic comparison of ISs locations. ISCompare requires two genomes to compare in an annotated Genbank format and a library of ISs. As an option, ISCompare can use all the ISs from ISFinder database (Siguier *et al.* 2006) for the automatic identification of DLISs. ISCompare algorithm uses the library of ISs (*i.e.*, nucleotide sequences) to look for ISs in the query and reference genomes. IR and DR information is not used to refine IS ends, thus it is recommended to use complete ISs with a high-quality annotation. Next, IS flanks are extracted, and used to compare the genomic context of the identified ISs. To determine DLISs, ISCompare only uses the genomic context, and thus, precise IS ends are not required. In addition, ISCompare algorithm was devised to work with draft bacterial genomes, consisting mostly of contigs with partial ISs on both ends. The output consists of several files, being the final-results table and the graphic report the most valuable ones. The final-results table reports all the differentially located IS candidates classified into 8 categories (Supplementary Figure S5). First, there are ISs classified as “DLIS” which are the ones with the highest degree of confidence. Second, there are categories that group ISs with flanks that correspond to other ISs, or genes occurring in multiple copies, and can produce false positives. And finally, there are hits that are discarded from the analysis because of several reasons (*i.e.*, flanks not found on the reference genome, flanks with query coverage below the cut-off values, and so on.). The graphic report file contains the genomic context of both the reference and query genomes, providing the means for a rapid inspection and verification of the results.

We analyzed the sensitivity and precision of ISCompare using artificially generated genomes with randomly generated insertions of a specified IS, showing that ISCompare performed very well, achieving a precision of 100% (92) and sensitivity of 94% (99.1) for the DLIS category (values in parentheses corresponds to VD category).

When we performed the analysis with real genomes, an increment in the number of false positive cases was observed (precision of 89% for *E. meliloti* strains). In that case, many of the false positive reports corresponded to DNA regions where the context of one side of the IS is correct, but the other is different, in general, because of genome rearrangements. Other false positives corresponded to regions containing very similar genes encoding integrases, which might be phage related, and in the case of *E. meliloti*, to group II introns. Interestingly, some of the locations reported presented a change of one IS by another. It should be noted that some IS might be absent on ISFinder database (*i.e.*, IS*Rm19*), in such cases the ISs present on the strains to analyze should be previously detected using tools like OASIS, ISQuest or ISEscan, manually curated and added to the IS.fasta file.

In the case of *E. meliloti*, we also determined the density of DLISs per replicon by comparing 26 genomes with the reference strain *E. meliloti* 1021. This analysis revealed a higher average number of DLISs per replicon (DLISs/Kb, Figure 3) for the symbiotic plasmid pSymA, as was expected in accordance with the higher plasticity associated with plasmids (L**ó**pez *et al.*^2019^). It should be noted, however, that to estimate the frequency of mobilization events on *E. meliloti*, further analysis discriminating by IS type and normalizing by the total number of ISs per replicon will be required.

A feature of ISCompare is that it can be also used to analyze the genomic context of transposons, phages or any other gene or DNA sequence in general. As a demonstration, in this work we used ISCompare to study the differentially located Group II introns in *E. meliloti*. For this comparison, the -S shift option, an exclusive running mode of ISCompare, greatly improved the detection of DLISs. However, although many new DLISs were identified (most of them associated to group II introns), some of the previously identified DLISs were missing (Supplementary Tables S6 and S7). It is important to notice that in the absence of genetic structures where the ISs are adjacent to sequences which occur multiple times on the genome, the use of -S option with values within the 1000–5000 base pairs range, could produce a decrease in the sensitivity.

In the case of *B. pertussis*, we analyzed strains with a Prn^-^ phenotype caused by IS*481* insertions in distinct positions of its coding sequence, which ISCompare was able to correctly identify.

From all the analyzed cases, our recommendation is to run ISCompare in normal mode, and if regions with many consecutive ISs or repeated sequences (such as group II introns) are present, run also in the shift (-S) mode. In both cases the DLIS results are very confident and when combined should improve the sensitivity of the program. Nevertheless, algorithms for the determination of DLISs can still be improved. When developing ISCompare, we wanted to make It simple enough to be able to run on computers with low resources. More complex algorithms using additional information such as the transposition mechanism, and IR and DR sequences, could improve the initial identification of ISs, and achieve better accuracy in the determination of DLISs.

In addition, there are exceptional cases that were not considered directly on ISCompare algorithm, some examples are ISs carrying passenger genes, or ISs with accessory genes with high similarity to non-IS related genes. In such cases, ISCompare results will be highly dependent on the IS library used. In the case of ISs carrying passenger genes (*e.g.*, IS*1* and IS*1595* family transporter ISs, Siguier *et al.* 2009), if the IS is not present in the library, but an IS closely related at the nucleotide level (above the *E*-value cut-off) is present, ISCompare will detect the shared sequence, which might lead to a false negative DLIS prediction. Those cases will often be tagged for manual verification and a careful analysis of the PDF report generated using the -p option is recommended. Furthermore, some of these situations might be solved by combining the normal and the shift modes.

In the case of ISs with accessory genes presenting high similarity to nonIS-related sequences (*e.g.*, IS*21 istB* gene, which product exhibits similarity to DnaA replication initiator protein; IS*200*/IS*605* and IS*607*, which often include a second orf, *tnpB*, encoding a protein with a helix-turn-helix domain, a central domain and C-terminal zinc finger; some ISs from the IS*91* family, which include a second orf related to the phage integrase family; and Tn*3* related ISs, which present a second gene encoding a site-specific recombinase; Siguier *et al.* 2014) ISCompare could erroneously identify such regions as ISs, however, it is most likely that those genes remain in the same genomic location and thus will not be reported as DLISs. To avoid such cases fine tuning of ISCompare parameters (*i.e.*, a more stringent *E*-value cut-off could be used) would be required.

Finally, we want to address the differences between ISCompare and ISSeeker. Both programs are based on the search of IS flanks from a query genome on a reference genome, however, there are several differences. First, ISSeeker presents the output in a different way, since it was thought for the fast and high-throughput comparison of the genome of many strains to a single reference genome. On the other hand, ISCompare was designed for pairwise IS location comparison. Second, the output table of ISSeeker shows the flanks of the detected ISs on the query genome, with the nearest position where those flanks align on the reference genome, and the position of all the ISs found on the reference genome. The identification of the DLISs can then be manually or graphically done by comparing those values. ISCompare, instead, generates a table containing the identified DLISs not requiring further analysis. Third, it should be noted that to get a complete idea of all the relocated ISs, ISSeeker should be run a second time using the reference genome as query. This is required since, for divergent enough strains, ISs could be present on genomic regions exclusively found in the reference strain. ISCompare focuses on identifying DLISs on conserved genomic regions, in the hope to find DLISs responsible for phenotype changes. Fourth, ISSeeker filtering steps are based on percent identity cut-offs, while ISCompare uses query coverage and *E*-value cut-offs. Fifth, ISseeker requires previous knowledge of an IS present in both genomes while ISCompare can be used to look for DLISs using a complete (or partial) database of ISs (such as ISFinder). Furthermore, using -I option, ISCompare will use ISFinder (Siguier *et al.* 2006) blast server to look for and download all the IS families found on the query and reference genomes. Last, although ISSeeker is faster (Table 2), ISCompare runs fast enough, can search simultaneously for thousands of ISs and outputs several tables with information about the ISs found on the query and reference genomes, cases of consecutive ISs which might yield false positives, a clear annotation of CDSs flanking the insertion sites, and a graphical PDF report showing the IS surroundings for manual verification of the DLIS status.

For all this, we think that ISCompare is a program that provides an easy and straightforward approach to look for DLISs between a pair of related bacterial genomes.
